# Inter-comparison of Mars Upper Atmosphere Neutral Density and Temperature Datasets from MAVEN

**DOI:** 10.1007/s11214-026-01302-w

**Published:** 2026-05-07

**Authors:** Nicholas Jones, J. Scott Evans, Sumedha Gupta, Sonal Jain, Marcin Pilinski, Shane W. Stone, Edward M. B. Thiemann, Nick Schneider, Shannon Curry

**Affiliations:** 1 Laboratory for Atmospheric and Space Physics, University of Colorado Boulder, 1234 Innovation Dr., Boulder, 80303 CO USA; 2 Computational Physics, Inc., 8001 Braddock Road, Springfield, 22151 VA USA; 3 Planetary Environments Laboratory, NASA Goddard Space Flight Center, 8800 Greenbelt Rd, Greenbelt, 20771 MD USA

**Keywords:** Mars atmosphere, Aeronomy, Dataset comparison, MAVEN

## Abstract

NASA’s Mars Atmosphere and Volatile EvolutioN spacecraft carries an extensive suite of instruments for characterizing the Mars upper atmosphere. Of these, the NGIMS, IUVS, and EUVM instruments produce datasets for CO_2_ density and neutral atmosphere temperature. The different instruments and retrieval methods utilized provide an expansive view of the Mars upper atmosphere. To make full use of the geophysical coverage offered by these datasets, we undertake a systematic comparison of the datasets to understand where they are different and how any biases between datasets can be removed. We conduct pairwise comparisons between datasets, binning the data by geophysical and forcing parameters, to develop adjustment factors that can be used to adjust the measured CO_2_ density and neutral temperature of one dataset to nominal agreement with another. The determined adjustment factors are reported for use by the wider Mars aeronomy community.

## Introduction

NASA’s Mars Atmosphere and Volatiles EvolutioN (MAVEN) spacecraft, arriving at Mars on 22 September 2014, has investigated the upper atmosphere of Mars for more than a decade utilizing a diverse suite of instruments and retrieval methods (Jakosky et al. 2015). The resulting datasets have been used to investigate MAVEN’s primary science questions related to current and past processes shaping the Martian atmosphere (see Lillis et al. 2015; Bougher et al. 2015a for details on the MAVEN science questions). For studies of the Mars neutral atmosphere, several MAVEN datasets are available that measure the density and temperature of neutral species in the upper atmosphere (e.g. Evans et al. 2015; Zurek et al. 2015; Stone et al. 2018; Thiemann et al. 2018; Gröller et al. 2018; Gupta et al. 2022; Evans et al. 2023). In this study, we seek to develop an understanding of the systematic differences that may exist between these different MAVEN measurements of the neutral atmosphere, focusing on datasets that retrieve CO_2_ density and neutral temperature in the upper atmosphere. By understanding what, if any, differences exist between the datasets, we aim to develop a means of systematically adjusting each dataset to remove these differences. The intention is to utilize these adjustments to allow the different neutral atmosphere datasets to be used together to enable greater coverage in the geophysical parameter space for studies of the Mars upper atmosphere. We conduct pair-wise comparisons of the Extreme UltraViolet Monitor (EUVM) solar occultation dataset, the Imaging UltraViolet Spectrograph (IUVS) Oxygen I 297 nm airglow emission dataset, the IUVS CO_2_^+^ Ultraviolet Doublet (UVD) airglow emission dataset, the IUVS stellar occultation dataset, and the Neutral Gas and Ion Mass Spectrometer (NGIMS, see Mahaffy et al. 2015b) in-situ measurement dataset. Each of theses datasets offers unique coverage of the geophysical parameter space, offering access to different combinations of latitude, local time, and altitude.

This paper is part of a series comparing datasets of the Martian upper atmosphere. As part of an effort to build the most complete understanding of the Mars upper atmosphere yet realized, we seek to link the MAVEN neutral atmosphere datasets to datasets produced by ESA’s Trace Gas Orbiter (TGO) mission (López-Valverde et al. 2018). TGO has been operating at Mars since October 2016, providing observations of the Martian atmosphere simultaneous, but often not co-located, with MAVEN observations. Alday et al. (2026, this collection) conduct a comparison of TGO neutral atmosphere datasets. To bridge the datasets of the two spacecraft, Thiemann et al. (2026, this collection) conduct a comparison of EUVM solar occultations with solar occultations taken by the Nadir and Occultation for MArs Discovery (NOMAD) instrument aboard TGO. Incorporating TGO datasets further augments the enhanced parameter space coverage that can be utilized by studies of the Mars upper atmosphere, especially with regards to contemporaneous measurements taken at different geophysical locations.

The remainder of the paper is organized as follows. In Sect. 2, we discuss the datasets utilized in this study, providing a general overview of their retrieval methods and the geophysical coverage offered by each. In Sect. 3, we discuss the inter-comparison process utilized to identify systematic differences between datasets where they exist and how each dataset can be adjusted to reference another dataset. In Sect. 4, we present the results of the inter-comparison process and provide the derived adjustment factors. In Sect. 5, we discuss in detail the results of the inter-comparison process and note interesting features revealed by the inter-comparison. In Sect. 6, we perform a brief analysis of the combined datasets pre- and post-adjustment to examine the effects of applying the adjustments to the datasets through different perspectives of the Martian thermosphere. In Sect. 7, we summarize the results of the dataset inter-comparison.

## Datasets

We utilize the following five MAVEN datasets for the inter-comparison:

- EUVM solar occultations dataset (Sect. 2.1)
- IUVS O I 297 nm airglow emissions dataset (Sect. 2.2.2)
- IUVS CO_2_^+^ Ultraviolet Doublet emissions dataset (Sect. 2.2.1)
- IUVS stellar occultations dataset (Sect. 2.2.3)
- NGIMS in-situ measurements (Sect. 2.3)

**Fig. 1:**
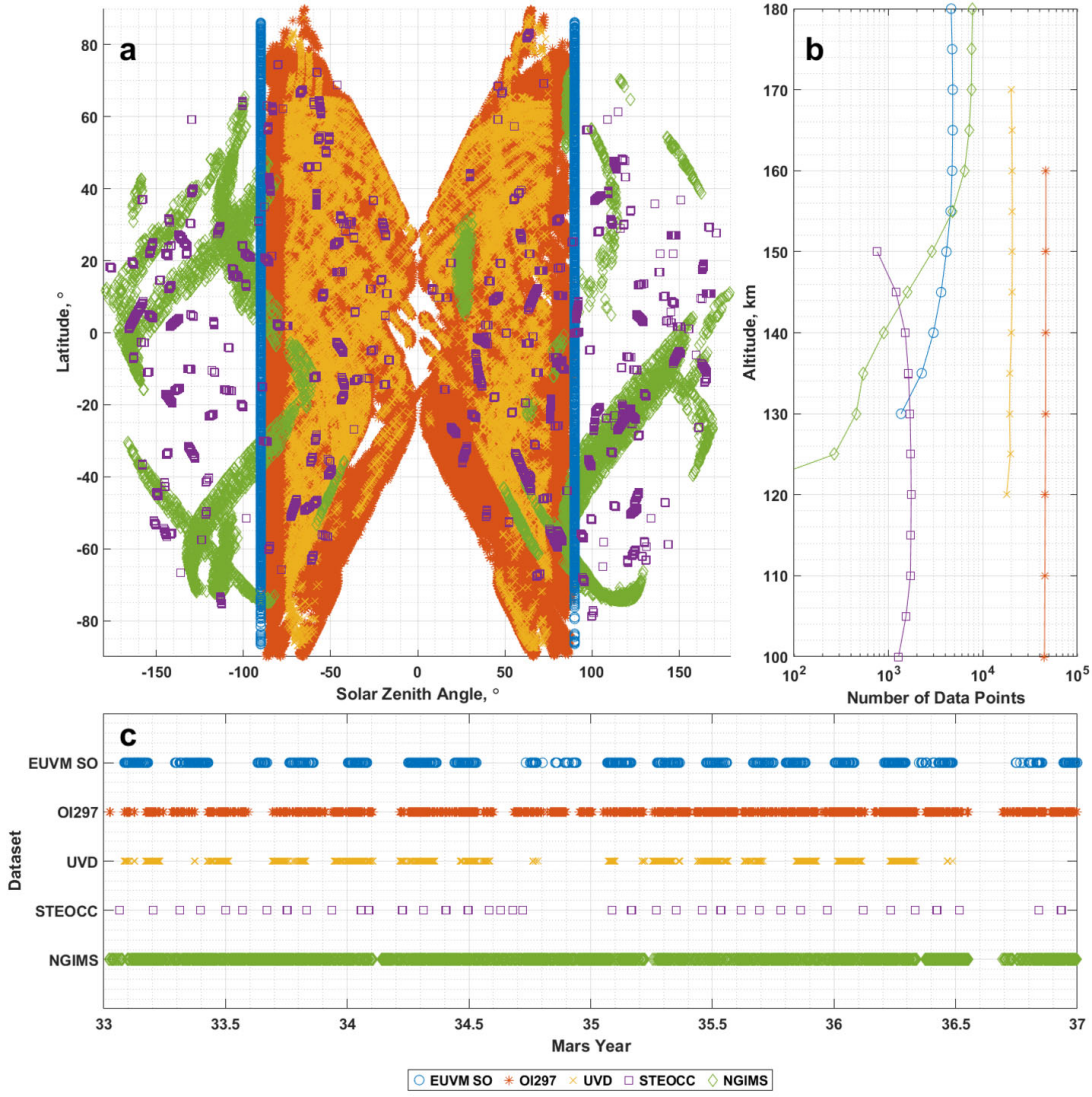
(a) Latitude and solar zenith angle coverage within the altitude range of 80 to 180 km for the inter-comparison datasets. The solar zenith angle is signed to discriminate between morning (negative SZA) and afternoon (positive SZA) measurements. (b) Number of data points for each dataset as a function of altitude. (c) Temporal sampling of the inter-comparison datasets, provided as a function of Mars Year. The horizontal axis (on this and other figures displaying plots against Mars Year, is divided into units of equal time. The plotted data in all 3 sub-figures span MY 33 through the end of MY 36

Each of the above datasets measures CO_2_ density and temperature profiles for the upper atmosphere of Mars utilizing different retrieval methods. The effect of utilizing different instruments and retrieval methods is that each dataset offers a unique perspective of the Mars atmosphere, as illustrated in Fig. 1. When plotting the distribution of data in latitude and solar zenith angle (SZA) in Fig. 1a, we utilized a signed SZA, where negative values correspond to morning side measurements, and positive values correspond to afternoon measurements. Signed SZA is used throughout the rest of this paper.

From Fig. 1a, which maps coverage in SZA and latitude for each dataset, we note that the IUVS airglow datasets (O I 297 nm emission and UVD emission) measure exclusively on the dayside of Mars, while the EUVM solar occultations are limited to the solar terminator. The IUVS stellar occultations and NGIMS measurements are able to measure on both the dayside and nightside of the planet. Figure 1b shows the altitude coverage offered by each instrument. Noting the logarithmic scale of the x-axis, we see that the most prolific datasets by number of data points are the IUVS airglow datasets, led by the O I 297 nm emission dataset. The number of data points contributed by the remaining three datasets varies with altitude. For this inter-comparison, we focus on the altitude range between 100 and 180 km, where the five datasets considered make their measurements and also overlap with other datasets. NGIMS continues to provide measurements above 180 km, and at low altitudes, the IUVS O I 297 nm dataset provides measurements down to 80 km. Figure 1c shows the temporal coverage of each dataset as a function of Mars year. The temporal coverage of each dataset depends not only on when MAVEN is appropriately positioned in its orbit for the desired measurement, but also on scheduling of observations between instruments (Jakosky et al. 2015). The result is that not all measurement opportunities can be taken advantage of, and the temporal coverage of an individual dataset is not as complete as it would be if operational conflicts between instruments did not exist.

For the purposes of this inter-comparison, we consider data from Mars year (MY) 33 through the end of MY 36, a full 4 Mars years spanning between June 2015 and December 2022. This allows us to consider multiple years of seasonal variation, a variety of dust loading conditions, and solar activity levels spanning from the declining phase of solar cycle 24 to the ramp up of solar cycle 25. For the purposes of the inter-comparison, the vertical profiles of CO_2_ density and temperature for each dataset are reported on a regular grid of altitudes between 100 and 180 km in 5 or 10 km increments where those datasets are available. The EUVM solar occultation, NGIMS, and IUVS stellar occultation datasets are interpolated onto a 5 km grid, while the IUVS UVD airglow dataset is natively reported at these altitudes. The IUVS O I 297 nm airglow dataset is natively reported at 10 km intervals. The altitude overlap region spans the Mars thermosphere, from near the Martian mesopause, which occurs at approximately 100 km, up to the Martian exobase, which occurs variably between 160 and 200 km (Bougher et al. 2017a). In the following sections, we describe each dataset briefly.

### EUVM Solar Occultations

Solar Extreme UltraViolet (EUV) light is absorbed by the Martian atmosphere between 100 and 220 km and is a primary energy input into the Martian thermosphere (Thiemann et al. 2018). The EUVM instrument onboard MAVEN measures the solar EUV spectrum in three spectral bands utilizing broadband radiometers. Channel A of EUVM measures the EUV spectrum between 0.1–3 nm and 17–22 nm, channel B measures between 0.1–7 nm, and channel C measures between 121–122 nm. The selection of these three bands allows EUVM to monitor emissions from multiple regions of the Sun, including the bright H I Lyman-$\alpha $ emission at 121.6 nm (Eparvier et al. 2015).

While the intent of the EUVM instrument was to allow accurate measurement of the solar EUV to better understand its role in influencing the dynamics of the upper atmosphere at Mars, Thiemann et al. (2018) realized additional scientifically relevant measurements using solar occultations measured by EUVM channel A during transitions of the MAVEN spacecraft into and out of eclipse. In the solar occultation technique, the absorption of solar EUV light as the instrument line-of-sight with the Sun transits the atmosphere is related to the amount of absorbing material in the column along the line-of-sight through application of Beer-Lambert’s law in the form

1 $$ I(z)=I_{0} \exp(-\sum _{i} N_{i}(z) \sigma _{i}) $$

where $I(z)$ is the irradiance measured at altitude $z$, $I_{0}$ is the reference irradiance measured with negligible absorption, $N_{i}$ is the column density of atmospheric constituent $i$, and $\sigma _{i}$ is the absorption cross section of that atmospheric constituent. As the instrument line-of-sight scans through the atmosphere, it measures a vertical profile of column density, which is then inverted through an Abel transform to find the number density. The retrieval performed in Thiemann et al. (2018) considers only CO_2_ as the sole atmospheric constituent. Applying hydrostatic equilibrium to the CO_2_ density profiles retrieves pressure profiles integrated from the top down. Applying the ideal gas law allows for the retrieval of temperature profiles, which are then fit to a Bates profile for final reporting (Bates 1959). Version 17 revision 02 of the EUVM solar occultation dataset reported in the level 4 EUVM data products is used for this study (Thiemann et al. 2025). We note that a multiplicative factor of 1.188 was applied to the CO_2_ densities of the solar occultation dataset to account for the known systematic error reported in Thiemann et al. (2018). Additional details and discussion of the dataset and its retrieval method are provided by Thiemann et al. (2018).

The geophysical parameter coverage realized by the EUVM solar occultation dataset is a result of many factors. An inherent aspect of the solar occultation technique is that observations are constrained to the dawn and dusk terminator, as seen in Fig. 1a. While this limits the local times that can be probed by this technique, it also introduces an inherent control on local time that can be useful for tracking long-term change within the Mars upper atmosphere. The unique observation geometry of EUVM solar occultations also allows it to observe locations away from where other instruments are performing their observations, helping to fill in the geophysical parameter space. The altitude coverage of the EUVM solar occultations is determined by a handful of factors. The first limit on altitude coverage is imposed by where significant absorption is occurring for the target spectral region. At the highest reaches of the atmosphere, where there is little material to absorb, and at the lowest measurement altitudes, where the signal is nearly extinguished, it is difficult to differentiate between changes in signal due to absorption and that due to noise. The altitude extent of measurements is also limited by known artifacts in the EUVM solar occultation dataset. At the topside, the assumption of a purely CO_2_ atmosphere causes absorption due to increasingly prominent atomic oxygen to be misattributed to CO_2_, leading to a relative over-count of CO_2_ at high altitudes (Thiemann et al. 2018). For this reason, the EUVM solar occultation dataset is limited to altitudes less than 180 km. As discussed in Thiemann et al. (2018), the systematic uncertainty of the retrieval increases at lower altitudes, so for this inter-comparison effort, we limit the solar occultation observations to altitudes greater than 130 km. Temporal coverage of the EUVM solar occultation dataset is determined by orbital and operational factors. Solar occultations can only occur when the MAVEN orbit takes the spacecraft into and out of eclipse, which occurs periodically as the orbit precesses around the planet. Solar occultation measurements can also only be made when EUVM is pointed at the Sun during these eclipse transitions. Due to the complex operations of the MAVEN spacecraft, which must coordinate other instrument observations and spacecraft activities that make demands on the spacecraft pointing, not all occultations can be observed by EUVM, reducing the size of the solar occultation dataset from ideal conditions.

### IUVS Datasets

The Imaging UltraViolet Spectrograph (IUVS) onboard the MAVEN spacecraft provides global 3-D density and temperature maps of the Martian atmosphere (McClintock et al. 2015; Jakosky et al. 2015). IUVS is an ultraviolet imaging spectrograph with a 10 x 0.06^∘^ slit containing occultation apertures at each end. The original MAVEN orbit was elliptical with apoapsis at ∼6200 km and periapsis nominally at an altitude of ∼175 km. Subsequent orbit adjustments over the course of the MAVEN mission did not impact the IUVS observations used in this study. Limb viewing allows for scans near periapsis as well as “inbound” and “outbound” segments of MAVEN’s orbit on either side of the periapse segment (Jain et al. 2015). IUVS measures Mars’ far ultraviolet (FUV) airglow between 110 and 190 nm at ∼0.6 nm resolution and middle ultraviolet (MUV) airglow between 180 and 340 nm at ∼1.2 nm resolution.

#### CO_2_^+^ UV Doublet

The main sources of CO_2_^+^ Ultraviolet Doublet (UVD; B${}^{2}\Pi _{u}^{+} \rightarrow X {}^{2}\Pi _{g}$) emission are photoionization and electron impact ionization of CO_2_ with photoionization of CO_2_ being the dominant source of emission at altitudes below ∼240 km where the peak of emission is ∼130 km (Fox and Dalgarno 1979; Jain and Bhardwaj 2012). Fluorescent scattering of sunlight by CO_2_^+^ is ignored as it contributes less than 10% to the total UVD emission at altitudes below ∼200 km (Fox and Dalgarno 1979; Fox 2004b,a; Jain and Bhardwaj 2012; Stiepen et al. 2015). Because UVD is emitted at 288–289 nm in a region of negligible pure absorption, accurate modeling of this transition is achievable within the combined uncertainties of the relevant photoabsorption and photoionization cross sections and the assumed solar irradiance. The Atmospheric Ultraviolet Radiance Integrated Code (AURIC; Strickland et al. 1999) first principles forward model is used to perform optimal estimation retrievals of CO_2_ densities from IUVS UVD emission profiles. Exospheric temperatures are derived by fitting an idealized Chapman function to altitude profiles of UVD column emission observed by MAVEN IUVS as described by Bougher et al. (2017b) and Lo et al. (2015) but with the generalized Chapman fitting formalism reported by Evans et al. (2020). The exospheric temperature (T_0_) is utilized as an upper boundary condition for deriving temperatures from retrieved CO_2_ densities using hydrostatic integration (Snowden et al. 2013; see Appendix A and Appendix B of Evans et al. 2023). In cases where the Chapman fit fails to converge ($< 1\%$), a value of 200 K is used for $T_{0}$. Version 13 revision 01 of the CO_2_^+^ UVD dataset reported in the Level 2 IUVS data products is used for this study.

#### O I 297 nm

Ultraviolet emissions from the oxygen O(^1^S) metastable state provide a valuable remote sensing signature of energy deposition, photochemistry, dynamics, and thermal structure in the Martian atmosphere and ionosphere. The production of metastable O(^1^S) atoms in the Martian atmosphere is dominated by photodissociation of CO_2_. Metastable O(^1^S) atoms radiatively relax by emitting photons at 297.2 nm (and other wavelengths, such as 557.7 nm; Gérard et al. 2020; Aoki et al. 2022). The oxygen 297.2 nm dayglow vertical profile exhibits a double-peaked structure with an upper emission peak (near 120 km) that is produced by solar extreme ultraviolet (EUV) photons, and a lower emission peak (near 85 km) that is produced primarily by solar Lyman-$\alpha $ photons. Observations of the O I 297.2 nm emission extend from about 80 km in the upper mesosphere to 160 km and above in the thermosphere, making this emission well-suited for retrieving composition, pressure, and temperature characteristics of the upper mesosphere and lower thermosphere of Mars. The AURIC model is used to perform optimal estimation retrievals of CO_2_ densities from IUVS O I 297.2 nm emission profiles. Hydrostatic integration (Snowden et al. 2013) of the CO_2_ density profiles is performed to obtain temperature profiles from 80–150 km. The same methodology described above for CO_2_^+^ UVD is used to obtain a Chapman fit derived exospheric temperature that is used as an upper boundary condition for the hydrostatic integration. In fact, the only difference between the retrieval methodologies for CO_2_^+^ UVD and O I 297 nm is in the forward model because of the different sources of emission. See Evans et al. (2023) for additional details on the retrieval methodology. The IUVS O I 297 nm airglow dataset used in this study is available at Evans et al. (2022). The version used in this work is dated 21 November 2023.

#### Stellar Occultations

Stellar occultation measures the stellar signal of a rising (egress) or setting (ingress) star along the line of sight (LOS) from the target (occulting star) to the spectrograph. As the stellar signal traverses the Martian atmosphere, it gets attenuated due to absorption by different atmospheric constituents. From these spectra, atmospheric transmission $T(\lambda , z)$ is calculated which is a function of wavelength $\lambda $ and tangent altitude $z$ along the LOS. It is the ratio of the unattenuated reference spectrum $I_{o}(\lambda )$ measured outside the atmosphere ($z>180\text{ km}$) and the attenuated spectrum $I (\lambda , z)$ modified due to absorption,

2 $$ T(\lambda , z) = \frac{I (\lambda , z)}{I_{o}(\lambda )} $$

IUVS executes stellar occultation observations in dedicated bimonthly campaigns, extending over 2–3 days and ∼12 orbits, observing 5–6 UV bright stars. For this study, only the far-ultraviolet (FUV) wavelength range 115–170 nm is considered as it accounts for the absorption by CO_2_ and O_2_ at atmospheric altitudes 80–160 km. This work also uses the improved stray light removal algorithm recently devised by Gupta et al. (2022) to process the dayside occultations, providing an unprecedented local time coverage to study diurnal variations in the Martian atmosphere as well as the climatology of its structure and composition.

The measured transmission spectrum is fit by the forward model that relates the LOS column abundances of CO_2_ (${N}_{\mathrm{CO}_{2}}$) and O_2_ (${N}_{ {\mathrm{O}_{2}}}$) by,

3 $$ T(\lambda ,z) = \exp\left (-\sum _{i}\sigma _{i}(\lambda )N_{i}(\lambda )\right ) $$

where $\sigma (\lambda )$ are the respective absorption cross sections for the $i$th species. The fitting procedure is based on a Levenberg-Marquardt algorithm (IDL-based MPFIT routines, see Markwardt 2009), which simultaneously adjusts $N_{\mathrm{CO}_{2}}$ and $N_{{\mathrm{O}_{2}}}$. The local density (number density) profiles of CO_2_ and O_2_ are retrieved from their respective column abundances by vertical inversion (onion peeling, see Quémerais et al. 2006). The obtained CO_2_ number density is furthermore used to calculate the temperature and pressure profiles by applying the constraint of hydrostatic equilibrium to it. Other details on retrieval methodology is provided in Gröller et al. (2018) and Gupta et al. (2022). The MAVEN/IUVS stellar occultation data used in this study can be accessed from Gupta (2024).

### NGIMS

In contrast to EUVM and IUVS, NGIMS obtains *in situ* measurements of the upper atmosphere at the location of the MAVEN spacecraft. Therefore, the MAVEN orbit and its precession about Mars in terms of local time and latitude determine the location of each NGIMS measurement and the dataset’s coverage of Mars. Science measurements are executed during each MAVEN periapse pass, from an altitude of roughly 500 km during the inbound portion of the pass, through a nominal periapsis of about 150–180 km altitude, to 500 km altitude on the outbound portion of the pass. Number densities are calculated from counts measured by the NGIMS detector in each mass per charge ($m/z$) channel between 2–150 amu with a mass resolution of 1 amu. In this work, we utilize NGIMS Level 1 (L1) export version 12 revision 1 data files and the same data are available publicly on the NASA Planetary Data System in MAVEN NGIMS Level 1b (L1b) version 9 revision 1 files (Elrod et al. 2014). In its closed source neutral mode, NGIMS measures the densities of CO_2_, Ar, N_2_, O, He, and H_2_ and the calculation of these quantities has been described previously (Stone et al. 2018, 2022). To measure the density of a neutral atmospheric species, NGIMS first ionizes the gas via electron impact, then filters ions by $m/z$, and finally counts the number of ions impacting the electron multiplier detector for each $m/z$ channel. The CO_2_ densities used here are constructed from count rates measured in $m/z$ channel 44, which corresponds to the molecular ion of the most abundant isotopologue of CO_2_, ${}^{12}\mathrm{C}{}^{16}\mathrm{O}_{2}{}^{+}$. If the instrument’s detector reaches saturation in $m/z=44$, then $m/z=45$ (corresponding to ${}^{13}\mathrm{C}{}^{16}\mathrm{O}_{2}{}^{+}$) is used as a proxy. During MAVEN’s week-long low-altitude excursions called Deep Dips (DDs), a second proxy is sometimes required when the detector reaches saturation in $m/z=45$. When necessary, $m/z=13$ (corresponding to the ${}^{13}\mathrm{C}^{+}$ fragment) is used as the second proxy. All count rates are corrected for detector dead time and background signal is removed. Corrections are then made to the measured densities for ram and instrumental effects. For a more detailed discussion of these corrections, see Stone et al. (2018, Sect. 2.1) and Stone et al. (2022, Sect. 2.1).

Neutral atmospheric temperature profiles are derived from the Ar density profile assuming hydrostatic equilibrium (Stone et al. 2018). As explained in Stone et al. (2018), the neutral temperature could feasibly be derived from the density profiles of CO_2_, Ar, or N_2_ and, in the region of the atmosphere sampled by NGIMS, we expect their temperatures to be equal. The Ar density is preferred for the calculation of the neutral temperature because Ar is chemically inert and data from $m/z=40$ exhibit minimal background signal. The selection of CO_2_ or N_2_ density profiles for the neutral temperature calculation would substantially decrease the size of the neutral temperature dataset used here due to higher signal-to-noise for these species and other instrumental effects previously described that can preclude the calculation of temperature (Stone et al. 2018, Sect. 2.3 and Fig. 8). On the outbound portion of each periapse pass, there is significant background signal for many species that arises due to the desorption of atmospheric gas that adsorbed onto the inner surfaces of the instrument during the inbound portion of the pass. However, this effect is quite small for Ar and other noble gases, which enables the use in this work of temperature profiles derived from Ar densities collected during the outbound portion of periapse passes. These outbound temperature profiles are derived in exactly the same manner as the inbound temperature profiles.

## Methodology

To conduct the inter-comparison of the MAVEN datasets, we first identify comparable measurements through binning the individual data points based on a set of geophysical parameters. The data is binned by altitude, latitude, solar zenith angle, Lyman-$\alpha $ irradiance, and a global dust index developed from the Laboratoire de Météorologie Dynamique (LMD) column dust optical depth climatology (Montabone et al. 2015, 2020). The data is sorted by altitude according to the interpolation scheme outlined in 2 and illustrated in Fig. 1b so that bins occur every 5 km, except for O I 297 nm data, which is reported every 10 km. Binning for the remaining four parameters is accomplished using a sliding window to construct the bins in each parameter. In latitude, bin centers are placed every 10° latitude between 80° S and 80° N with 20° bin width. A similar scheme is used to form the solar zenith angle bins, but the bin centers are placed between −180° and 170° while using the same bin spacing and width. Solar zenith angle is preferred over binning in local solar time as the terminator wanders in local solar time over the course of a Mars year. We chose to utilize binning by Lyman-$\alpha $ irradiance and dust activity in order to capture drivers of the Mars upper atmosphere state from above and below. Solar EUV forcing has been demonstrated to be a significant driver of thermospheric temperature at Mars (Thiemann et al. 2018; Jain et al. 2023). Dust activity in the Martian lower atmosphere has been shown to be related to significant changes in the structure, composition, and dynamics of the upper atmosphere (Liu et al. 2018; Elrod et al. 2020; Yiğit et al. 2021; Yoshida et al. 2021).

**Fig. 2:**
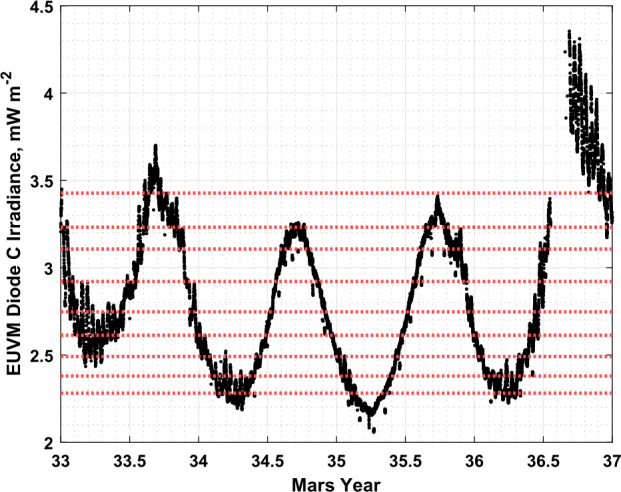
Orbit average EUVM channel C measurements of Lyman-$\alpha $ irradiance, plotted in black (Eparvier et al. 2015). Red dashed lines indicate every tenth percentile of the data from the 10th to 90th percentile. The data gap just after MY 36.5 is due to a MAVEN safe mode event, which precluded science measurements during this period

To bin by the Lyman-$\alpha $ irradiance, we interpolate the orbit-averaged EUVM channel C irradiance measurement, reported in the EUVM version 17 revision 2 level 2b data product, in time to each data points. As discussed in Sect. 2.1, channel C of EUVM measures between 121 and 122 nm and captures the bright Lyman-$\alpha $ emission from the Sun. Binning by Lyman-$\alpha $ allows us to capture forcing from above the atmosphere by solar EUV irradiance. The orbit-averaged EUVM diode C record for MY 33 through MY 36 is shown in Fig. 2. From Fig. 2, the strong seasonal variation in EUV irradiance due to the high eccentricity of the Mars orbit over the course of the Mars year is apparent, along with the longer-term modulation by the progression of the solar cycle. To promote an equitable distribution of data points between bins, a percentile-based binning scheme is employed for sorting the data by Lyman-$\alpha $ irradiance. Figure 2 shows every tenth percentile of the irradiance data as red dashed lines, which double as the Lyman-$\alpha $ bin centers. Each bin extends to the neighboring bin center, so that each bin covers 20% of the Lyman-$\alpha $ irradiance range. The highest and lowest bins extend up to the maximum and minimum irradiance measurement for MY 33 through MY 36, respectively.

**Fig. 3:**
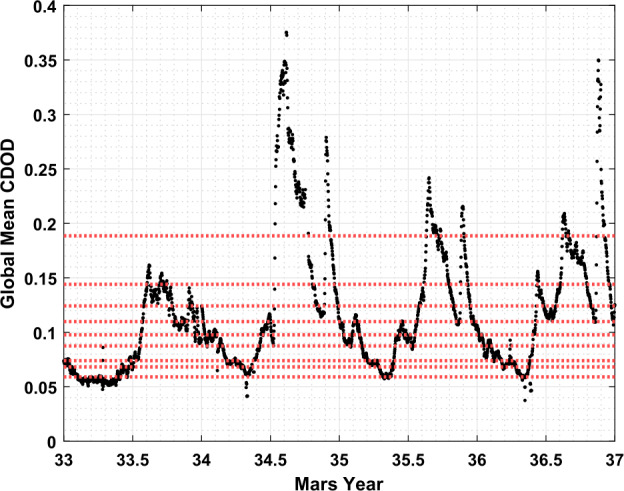
Global mean column dust optical depth (CDOD) time series for MY 33 through 36. See text for how this was developed from the LMD Mars dust climatology dataset (Montabone et al. 2015, 2020). The red dashed lines indicate every 10th percentile of the data from the 10th to 90th percentile

To capture atmospheric conditions forcing the Martian thermosphere from below, we developed a dust activity index from the LMD dust optical depth climatology maps (Montabone et al. 2015, 2020). This is accomplished by calculating the global mean of the daily column dust optical depth (CDOD) maps, weighted by the reported uncertainty, for each Mars Year and Sol where these maps are reported. The available dust maps from the LMD database span MY 33 through MY 36 at the time of writing. We utilize the irregularly gridded 9.3 μm absorption column dust optical depth normalized to the 610 Pa pressure level data product provided within the LMD dataset to develop the global mean CDOD parameter as these data are based solely on observations and best represent the actual dust activity that occurred at the time of measurement (as opposed to the complete-coverage, reconstructed maps that are also provided, but suggested for use in Mars climate modeling). The LMD dataset reports spatial resolution of 6° latitude by 5° longitude for MY 33 through MY 36. For further details on the specific LMD dust climatology data product used, the reader is referred to the relevant publications (Montabone et al. 2015, 2020). The resulting time series of global mean CDOD is plotted in Fig. 3. The times series of global mean CDOD data generated by this procedure are then interpolated for the specific Mars Year and solar longitude at which density and temperature measurements occur. The global mean CDOD bins are formed using the same percentile-based binning scheme as for the Lyman-$\alpha $ bins, discussed above.

To ensure the robustness of the comparison, we set rules on the binned data to eliminate poorly populated bins. We require at least 20 data points from each dataset per bin for that bin to be included in the analysis, and at least 10 valid bins to complete the inter-comparison. Once the data has been binned, the mean and standard deviation of the density and temperature are calculated for each bin, weighting the measurements by their reported uncertainty. The binned data means and standard deviations are then used for the inter-comparison.

A linear fit on the $\log_{10}$ of the CO_2_ densities is performed through reduced major axis (RMA) regression on

4 $$ \log_{10}(\rho _{2}) = m \log_{10}(\rho _{1}) + b $$

where $\rho _{1}$ and $\rho _{2}$ are the mean densities of the two datasets being compared for each geophysical parameter bin, $m$ is the slope of the fit, and $b$ is the intercept. We utilize RMA regression to allow for handling uncertainties on both datasets and provide equivalency between comparing dataset 1 to dataset 2 and dataset 2 to dataset 1 (Draper 1991). Equation (4) is expressed equivalently as,

5 $$ \rho _{2} = 10^{b} \rho _{1}^{m} $$

For temperature, a linear fit is performed on the temperatures through RMA regression on

6 $$ T_{2} = m T_{1} + b $$

where $T_{1}$ and $T_{2}$ are the mean temperatures of the two datasets being compared for each geophysical parameter bin, $m$ is the slope of the fit, and $b$ is the intercept. We also calculated the difference between the means of the binned temperature as

7 $$ \Delta T = T_{2} - T_{1} $$

where $T_{1}$ and $T_{2}$ are as defined for Eq. (6). Based on the degree of correlation observed in the temperature inter-comparison, either the RMA regression on the temperature or the median of the temperature differences, $\Delta T$, calculated by Eq. (7) will be considered as the preferred method of comparing temperature. When correlation is low between dataset temperatures and the calculated fit poorly describes the data, as determined by calculating the adjusted $R^{2}$ coefficient for the fit, the median temperature difference will be the preferred comparison metric. Otherwise, the RMA regression will be used to compare temperatures. The RMA regression on the $\log_{10}$ CO_2_ densities is always used to compare the CO_2_ densities.

Equation (4), Eq. (6), and Eq. (7) are calculated twice for the binned data. The first application of these equations is used to identify outlier bins. This is done for the RMA regressions of Eq. (4) and Eq. (6) by calculating the residuals of the fit (fit value minus mean bin value for dataset 2) for each bin, calculating the standard deviation of the residuals, and marking any bin outside of 3 standard deviations as outliers. When the median temperature difference is the preferred method of comparing the binned temperatures, data outside 3 standard deviations of the median temperature difference are marked as outliers. The RMA regressions and median temperature difference are then recalculated, excluding the identified outlier bins. The residuals and temperature difference spread can be used to further investigate the inter-comparison of the datasets for structure and correlations with geophysical parameters, as will be done in the following sections. We also investigate whether the inter-comparison suggests nominal agreement between the datasets.

Once the fit parameters are calculated for Eq. (4) and Eq. (6), along with the median temperature difference where that is preferred, we can adjust each dataset to “reference” the other for combined analyses. To fully enable this, however, we must propagate the uncertainty of the adjustment methods into the adjusted dataset.

### Uncertainty Propagation

Within each bin, the variability around the mean CO_2_ density and temperature are much greater than the calculated uncertainty on either mean, and so the standard deviation of the data within each bin is taken as the measure of uncertainty on the means. Uncertainty on the RMA regression slopes and intercepts are estimated using a bootstrapping procedure (Efron 1982). In this procedure, the binned data is randomly resampled with replacement, preserving the total number of bins. The slope and intercept are calculated for this new dataset. This is repeated 10,000 times to build a statistical distribution of the slope and intercept. The standard deviation of each parameter and the covariance between the slope and intercept are calculated based on these resampled datasets. The uncertainty on the median temperature difference is taken as the standard deviation of the binned temperature differences for that comparison.

While the RMA regression is performed on the $log_{10}$ CO_2_ densities in the form of Eq. (4), it is more useful to utilize Eq. (5) to adjust the datasets. To calculate the uncertainty of the adjusted dataset, we apply

8 $$ \sigma _{\rho _{2}}^{2} = 10^{2 b} \rho _{1}^{2 m} ( \frac{m^{2}}{\rho _{1}^{2}} \sigma _{\rho _{1}} + \ln(\rho _{1})^{2} \sigma _{m}^{2} + \ln(10)^{2} \sigma _{b}^{2} + 2 \ln(m) ln(10) \sigma _{mb}) $$

where $\rho _{1}$ and $\rho _{2}$ are the CO_2_ densities for dataset 1 and 2 of the inter-comparison with measurement uncertainties $\sigma _{\rho _{1}}$ and $\sigma _{\rho _{2}}$ respectively, $m$ is the regression slope with uncertainty $\sigma _{m}$, $b$ is the regression intercept with uncertainty $\sigma _{b}$, and $\sigma _{mb}$ is the covariance between the slope and intercept. Applying uncertainty propagation to the temperature regression yields

9 $$ \sigma _{T_{2}}^{2} = m^{2} \sigma _{T_{1}}^{2} + T_{1}^{2} \sigma _{m}^{2} + \sigma _{b}^{2} + 2 T_{1} \sigma _{mb} $$

where $T_{2}$ and $T_{1}$ are the temperatures for dataset 1 and 2 of the inter-comparison with measurement uncertainties $\sigma _{T_{1}}$ and $\sigma _{T_{2}}$ respectively, with the slope, intercept, their uncertainties, and the slope-intercept covariance defined as for Eq. (8). When the median temperature difference is the preferred temperature adjustment method, uncertainty is propagated when adjusting datasets through

10 $$ \sigma _{T_{2}}^{2} = \sigma _{T_{1}}^{2} + \sigma _{\Delta T}^{2} $$

where the temperature uncertainties are defined as for Eq. (9) and $\sigma _{\Delta T}$ is the standard deviation of the binned temperature differences.

## Results

**Fig. 4:**
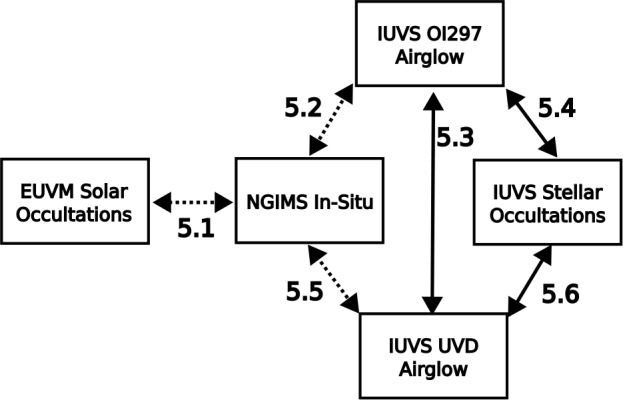
Diagram mapping adjustment “routes” following the successful comparisons summarized in Tables 1 and 2. A solid line indicates temperatures can be adjusted with a linear fit, while a dashed arrow indicates temperatures are limited only to adjustment by the median temperature difference. The $\log_{10}$ density regression adjustment can be applied along any arrow. Section numbers are placed on each arrow to indicate where the relevant inter-comparison is discussed in the text

Pairwise comparisons were made between the datasets listed in Sect. 2 according to the methodology laid out in Sect. 3. The resulting adjustment parameters are outlined in Table 1 for the CO_2_ density comparison and in Table 2 for the temperature comparison. Not all pairwise comparisons could be completed due to a lack of overlap in the binning parameters. This restricts the EUVM solar occultation dataset to comparison with only the NGIMS dataset. Because the method of solar occultations locks the measurements to the terminator, EUVM solar occultations cannot be compared with the IUVS airglow datasets, which are limited to the dayside (i.e., SZA $<75^{\circ}$). The IUVS stellar occultation dataset and EUVM solar occultation dataset lack overlap in the binning parameters needed to build enough valid bins to conduct the comparison. The IUVS stellar occultations cannot be compared with the NGIMS dataset for the same reason. Based on the comparisons that could be successfully completed, Fig. 4 maps out the “routes” through which each dataset can be adjusted to another. Following these routes, each dataset can be adjusted to a chosen reference dataset for combined analysis.

**Table 1:**
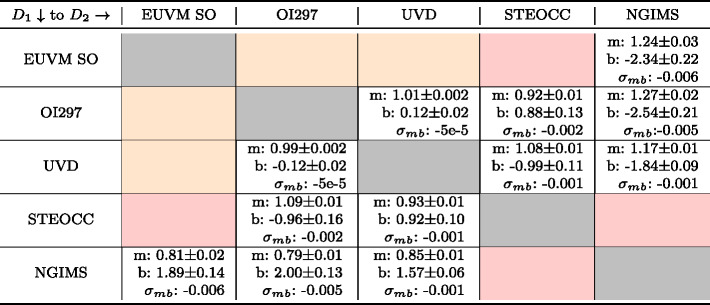
Summary of the slope and intercept along with their $1\sigma $ variations determined for each density inter-comparison for which the analysis could be conducted. $m$ is the slope, $b$ is the intercept, and $\sigma _{mb}$ is the slope-intercept covariance for the regression. Orange shaded cells indicate a lack of overlap in geophysical parameters, preventing a comparison between the two datasets using our methods. Red shaded cells indicate that, while overlap exists, not enough data points were available to complete the comparison. The row indicates the starting dataset, corresponding to subscript 1 terms in Eq. (4), and the column indicates the dataset to adjust to, corresponding to subscript 2 terms in Eq. (4)

**Table 2:**
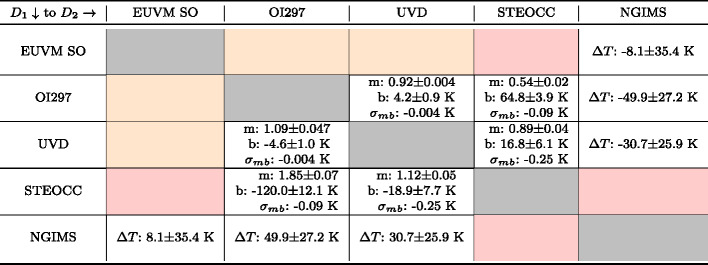
Summary of the temperature inter-comparison adjustment parameters along with their $1\sigma $ variations determined for each inter-comparison for which the analysis could be conducted. $m$ is the slope, $b$ is the intercept, and $\sigma _{mb}$ is the slope-intercept covariance for the regression. $\Delta T$ is the median temperature difference, provided where the RMA regression does not well-describe the data. Orange shaded cells indicate a lack of overlap in geophysical parameters, preventing a comparison between the two datasets using our methods. Red shaded cells indicate that, while overlap exists, not enough data points are available to complete the analysis. The row indicates the starting dataset, corresponding to subscript 1 terms in Eqs. (6) and (7), and the column indicates the dataset to convert to, corresponding to subscript 2 terms in Eqs. (6) and (7)

**Fig. 5:**
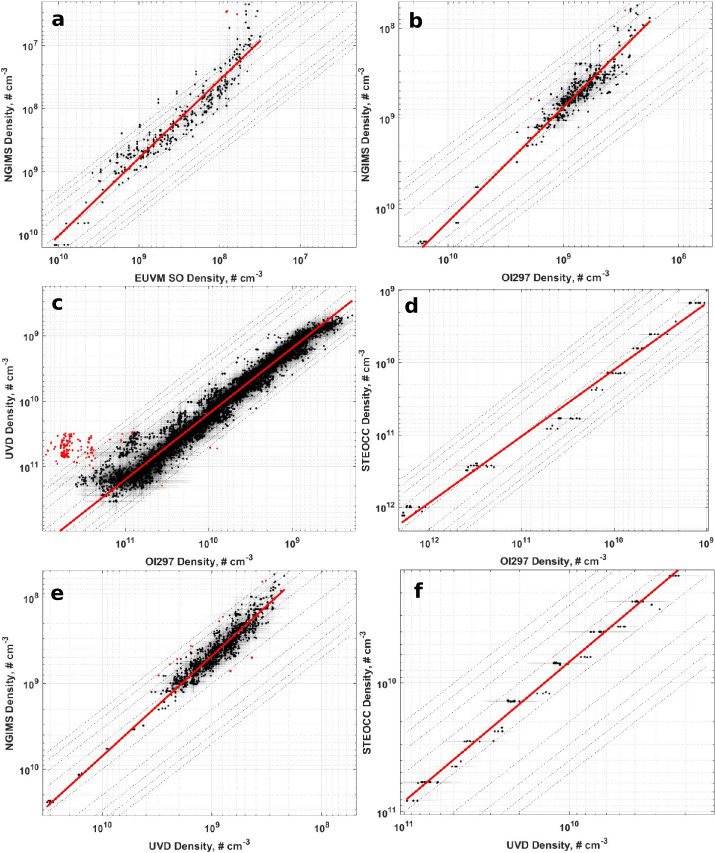
CO_2_ density inter-comparison plots. The binned densities are plotted in black along with their $1\sigma $ standard deviations as transparent errorbars. Points identified as outliers are marked in red. The red line indicates the RMA regression line. Note the reversed axes

**Fig. 6:**
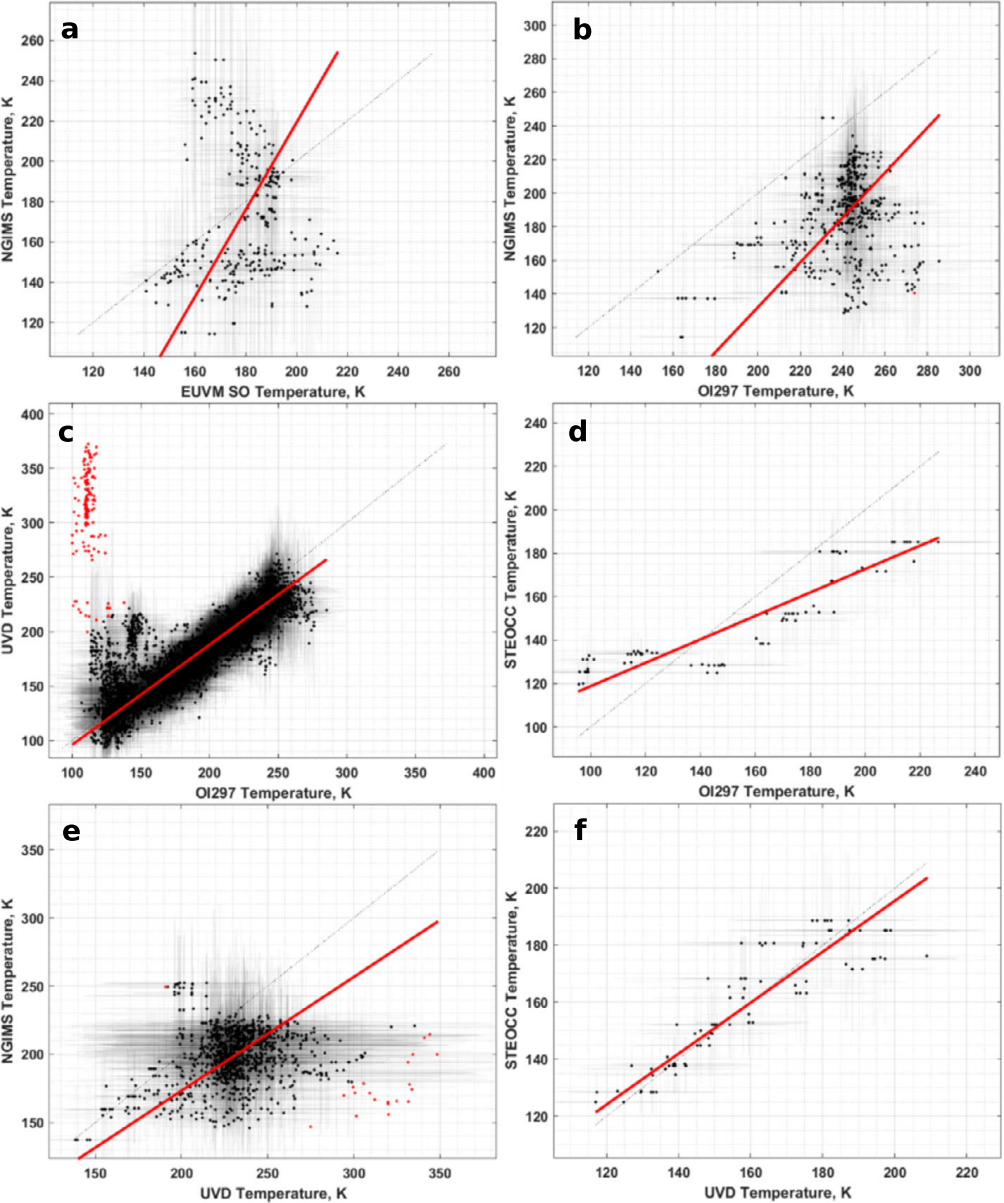
Temperature inter-comparison plots. The binned temperatures are plotted in black along with their $1\sigma $ standard deviations as transparent errorbars. Points identified as outliers are marked in red. The red line indicates the RMA regression line

In Figs. 5 and 6 we present graphical representations of the CO_2_ density and temperature inter-comparisons between datasets. Each subplot shows one of the pairwise comparisons, plots the binned data, CO_2_ density or temperature as appropriate, identifies the outlier data, and shows the calculated RMA regression. Table 3 contains the Pearson correlation coefficients, adjusted $R^{2}$ values for the RMA regressions, and the number of bins used for each comparison. In general, the correlations for the CO_2_ density comparisons are high with similarly high adjusted $R^{2}$ values. In contrast, correlations between temperatures are lower, especially for comparisons with NGIMS temperatures. Figures 6a, 6b, and 6e demonstrate that EUVM solar occultation, IUVS O I 297 nm airglow and IUVS UVD airglow temperatures are not well correlated with NGIMS temperatures. There does not exist a statistically significant correlation at the $95\%$ confidence level between EUVM solar occultations and NGIMS, and the correlations between IUVS O I 297 nm and UVD airglow temperatures with NGIMS are low. The negative adjusted $R^{2}$ values reported in Table 2 for the comparison of these three datasets with NGIMS suggest the mean is a better descriptor of the data than the RMA regression. In these cases, we prefer to use the median temperature differences reported in Table 2 for adjusting the datasets to NGIMS levels. The temperatures for the IUVS datasets, O I 297 nm airglow, UVD airglow, and stellar occultations, are well correlated and therefore can utilize the RMA regression for adjusting the temperature datasets.

**Table 3:** Inter-comparison Pearson correlation coefficient, RMA regression adjusted $R^{2}$ value, and number of bins used. Correlation coefficients are only recorded where they are statistically significant at the 95$\%$ confidence level

	CO_2_ Density			Temperature		
Comparison	Correlation	Adj. $R^{2}$	$N_{\mathrm{Bin}}$	Correlation	Adj. $R^{2}$	$N_{\mathrm{Bin}}$
EUVM SO to NGIMS	0.94	0.89	352		−0.99	288
OI297 to NGIMS	0.94	0.88	471	0.22	−0.54	490
OI297 to UVD	0.97	0.97	8911	0.67	0.81	8950
OI297 to STEOCC	0.99	0.98	78	0.90	0.80	78
UVD to NGIMS	0.95	0.91	1192	0.16	−0.49	1222
UVD to STEOCC	0.99	0.99	91	0.90	0.79	91

In the next section, we examine the results of the inter-comparisons more deeply, in addition to looking at the residuals of the RMA regressions with respect to different geophysical parameters. For the case of the temperature comparisons with NGIMS, we examine the binned temperature differences.

## Discussion

When considering the residuals plotted against one of the five binning parameters (altitude, latitude, SZA, Lyman-$\alpha $ irradiance, and global average CDOD), we calculate the average and standard deviation of the residuals for that parameter bin. For the binning parameters of altitude, Lyman-$\alpha $ irradiance, and global average CDOD, we also calculate the Pearson correlation coefficient of that parameter with the residuals. These are summarized in Table 4. Most of these correlations are small, with only five of them having a magnitude greater than 0.5.

**Table 4:** Inter-comparison Pearson correlation coefficients of the CO_2_ density and temperature RMA regression residuals with altitude, Lyman-$\alpha $ irradiance, and global average CDOD. For NGIMS temperature comparisons, we look at correlations with the binned temperature differences. Correlation coefficients are only recorded where they are statistically significant at the 95$\%$ confidence level

	CO_2_ Density			Temperature		
Comparison	Alt	Lyman-*α*	Avg. CDOD	Alt	Lyman-*α*	Avg. CDOD
EUVM SO to NGIMS		0.11	0.20	0.20	0.49	0.51
OI297 to NGIMS		0.24		−0.16	0.41	0.19
OI297 to UVD	0.17	0.37	0.28	0.09	−0.36	−0.31
OI297 to STEOCC						
UVD to NGIMS	−0.15	−0.08		−0.27	0.42	0.45
UVD to STEOCC		−0.57	−0.72		0.57	0.55

### EUVM Solar Occultations to NGIMS

**Fig. 7:**
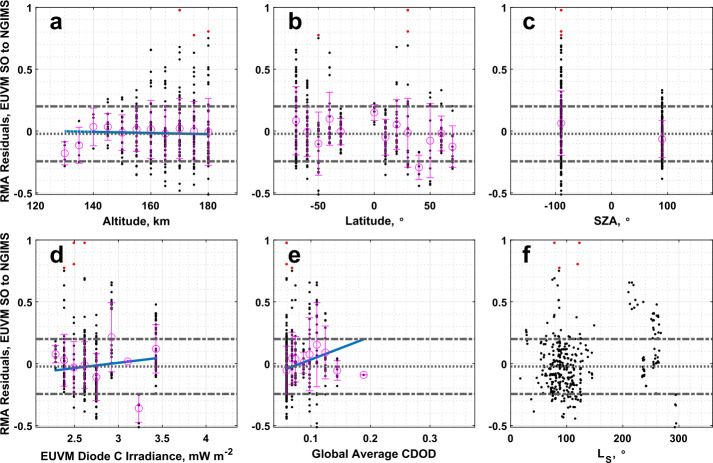
Residuals of the RMA regression between EUVM solar occultations and NGIMS CO_2_ density datasets plotted against (a) altitude, (b) latitude, (c) SZA, (d) EUVM diode C irradiance, (e) global average CDOD, and (f) $L_{\mathrm{S}}$. Red points indicate outliers. Magenta points with error bars indicate bin averages for the binning parameters and their standard deviations. The blue line indicates a linear fit to the binned data. Positive residuals indicate EUVM solar occultation data is measuring below the best-fit line

Inspection of Fig. 5a shows that EUVM solar occultation and NGIMS densities correlate well with one another. From Table 1, we note that the slope and intercept are both outside the $3\sigma $ level of nominal equality between the two datasets. If the datasets were in agreement, we would expect a slope equal to 1 and an intercept equal to 0. We observe greater disagreement at low densities. We also observe from Fig. 7a and 7c, that the highest residual data points are clustered at high altitudes and at the dawn terminator. This may be indicative of the effects of a known artifact in the EUVM solar occultation dataset. In Sect. 2.1, it was discussed that in the retrieval for the solar occultations, CO_2_ is the only atmospheric constituent considered. However, at higher altitudes, O becomes an increasingly significant component of the Mars atmosphere, before becoming the dominant species above approximately 200 km (Nier and McElroy 1977; Mahaffy et al. 2015a; Bougher et al. 2015b). As a result, EUV extinction that in reality is due to O is instead attributed to CO_2_ in the solar occultation retrieval algorithm, possibly leading to the overestimation of CO_2_ density by EUVM solar occultations at high altitudes. This artifact is discussed in further detail in Thiemann et al. (2018). The concentration of the highest residual data points on the dawn terminator could be an indication of an enhancement of lighter species in the Mars atmosphere due to transport from the dayside to nightside, as was observed in Stone et al. (2022). This enhancement occurs prominently for O which, as discussed previously, is known to interfere with EUVM solar occultation measurements at higher altitudes, where higher residuals of the inter-comparison are also seen to occur. Only weak correlation between the RMA regression residuals and Lyman-$\alpha $ irradiance or global average CDOD is detected at the 95% confidence level, and there does not appear to be any latitudinal structure to the residuals as plotted in Fig. 7b.

**Fig. 8:**
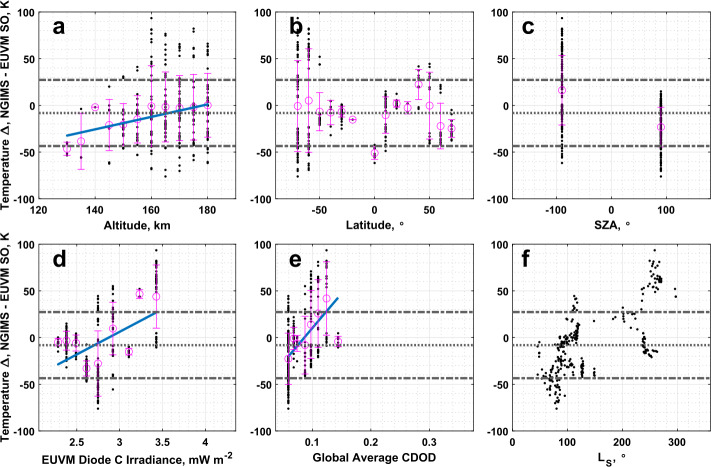
Binned temperature differences between EUVM solar occultations and NGIMS temperature datasets plotted against (a) altitude, (b) latitude, (c) SZA, (d) EUVM diode C irradiance, (e) global average CDOD, and (f) $L_{\mathrm{S}}$. Red points indicate outliers. Magenta points with error bars indicate bin averages for the binning parameters and their standard deviations. The blue line indicates a linear fit to the binned data

As noted in Sect. 4, the NGIMS temperatures are not well correlated with the other datasets. From Table 2, NGIMS measures slightly cooler temperatures than EUVM solar occultations, with a large spread that is well within 1 standard deviation of a 0 K difference. While no data points are identified as outliers for the comparison of EUVM solar occultation and NGIMS temperatures, we do note an apparent dependence on season. As reported in Table 4 and illustrated in Fig. 8d and 8e, there is a statistically significant correlation for the temperature difference between two datasets with Lyman-$\alpha $ irradiance and global average CDOD. We note from Fig. 8f, which plots the temperature differences as a function of the mean Martian solar longitude ($L_{\mathrm{S}}$) of the measurements composing each bin, that the highest positive temperature differences occur near perihelion at an $L_{\mathrm{S}}$ of approximately 270°. From Fig. 8b, we note that the largest positive temperature differences are concentrated in the southern hemisphere. Combined with the high correlation of the temperatures with the global average CDOD, the concentration of the highest temperature differences in the southern hemisphere during southern summer, where we would expect the peak dust activity from seasonal dust storms, may indicate an influence of dust activity on the temperature retrieval from either NGIMS or EUVM solar occultation densities. The correlation with Lyman-$\alpha $ irradiance, then, is also expected, as the highest EUV irradiance will occur near perihelion when dust activity is also high.

### IUVS O I 297 nm Airglow to NGIMS

**Fig. 9:**
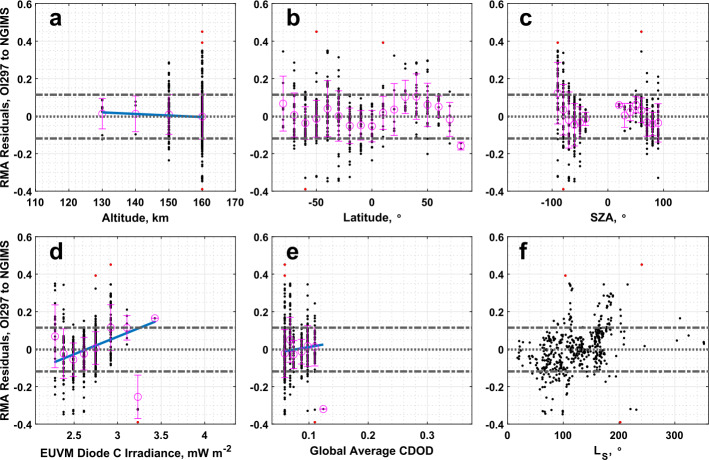
Residuals of the RMA regression between IUVS O I 297 nm airglow and NGIMS CO_2_ density datasets plotted against (a) altitude, (b) latitude, (c) SZA, (d) EUVM diode C irradiance, (e) global average CDOD, and (f) $L_{\mathrm{S}}$. Red points indicate outliers. Magenta points with error bars indicate bin averages for the binning parameters and their standard deviations. The blue line indicates a linear fit to the binned data. Positive residuals indicate IUVS O I 297 airglow data is measuring below the best-fit line

Like with the comparison of CO_2_ density between EUVM solar occultations and NGIMS, the densities measured by IUVS O I 297 nm airglow and NGIMS are well correlated. From Table 1, we see that the RMA regression slope and intercept are significantly different from agreement conditions. A small, but statistically significant correlation of the RMA regression residuals with Lyman-$\alpha $ irradiance is reported in Table 4. From Fig. 9c, we note that the high residual data points occur near the dawn terminator.

**Fig. 10:**
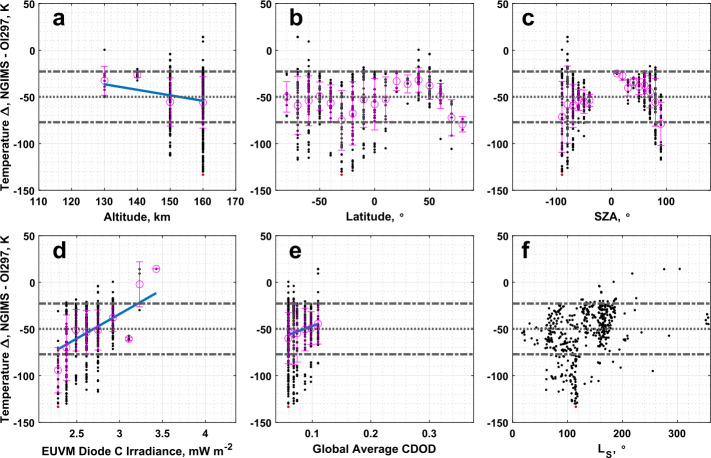
Binned temperature differences between IUVS O I 297 nm airglow and NGIMS temperature datasets plotted against (a) altitude, (b) latitude, (c) SZA, (d) EUVM diode C irradiance, (e) global average CDOD, and (f) $L_{\mathrm{S}}$. Red points indicate outliers. Magenta points with error bars indicate bin averages for the binning parameters and their standard deviations. The blue line indicates a linear fit to the binned data

O I 297 nm airglow temperatures are poorly correlated with NGIMS temperatures, with a correlation coefficient of only 0.22. The O I 297 nm airglow temperatures are also much warmer compared to NGIMS, but the large spread in the temperature differences keeps the median temperature difference within 2 standard deviations of 0 K. Low, but statistically significant correlations are reported between the binned temperature differences with altitude, Lyman-$\alpha $ irradiance, and global average CDOD, with the Lyman-$\alpha $ irradiance correlation being the highest at 0.41. The largest temperature differences appear to occur near the terminator region, as illustrated in Fig. 10c.

### IUVS O I 297 nm Airglow to IUVS UVD Airglow

**Fig. 11:**
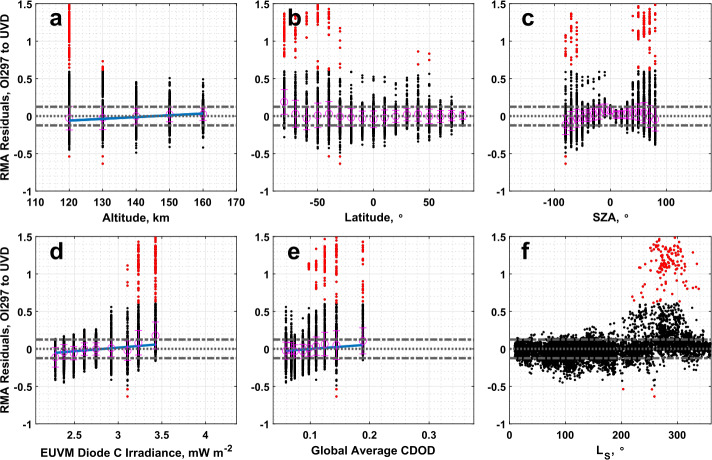
Residuals of the RMA regression between IUVS O I 297 nm airglow and IUVS UVD airglow CO_2_ density datasets plotted against (a) altitude, (b) latitude, (c) SZA, (d) EUVM diode C irradiance, (e) global average CDOD, and (f) $L_{\mathrm{S}}$. Red points indicate outliers. Magenta points with error bars indicate bin averages for the binning parameters and their standard deviations. The blue line indicates a linear fit to the binned data. Positive residuals indicate IUVS O I 297 airglow data is measuring below the best-fit line

**Fig. 12:**
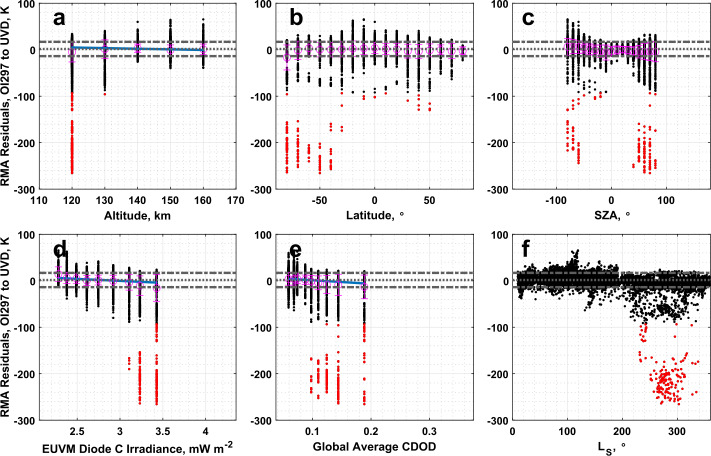
Residuals of the RMA regression between IUVS O I 297 nm airglow and IUVS UVD airglow temperature datasets plotted against (a) altitude, (b) latitude, (c) SZA, (d) EUVM diode C irradiance, (e) global average CDOD, and (f) $L_{\mathrm{S}}$. Red points indicate outliers. Magenta points with error bars indicate bin averages for the binning parameters and their standard deviations. The blue line indicates a linear fit to the binned data. Positive residuals indicate IUVS O I 297 airglow data is measuring below the best-fit line

The extensive overlap between the IUVS airglow datasets enables an extensive comparison and provides a relatively high degree of certainty on the adjustment parameters reported in Tables 1 and 2. From Table 1, the slope of the RMA regression is greater than $3\sigma $ from 1, while the intercept is not significantly different from 0. While the majority of the airglow CO_2_ density data is well correlated, we do note a cluster of outlier data points at high densities, where a subset of the UVD airglow dataset appears to stop responding to increasing density reported by the O I 297 nm airglow dataset, just below $10^{11}\text{ cm}^{-3}$ CO_2_ density. Examining the RMA regression residuals in Fig. 11, we note that these outliers are concentrated at low altitudes, the southern hemisphere, near the terminator (high SZA), at perihelion, and during high dust activity. Figures 11e and 12e illustrate that the outliers exist exclusively at higher global average CDOD. The UVD data below 130 km are prone to an a priori bias when the peak of UVD emission is higher in altitude than average (e.g., large SZA, increased dust activity). Since there is limited information content in the UVD emission profile a few CO_2_ scale heights below the peak of emission (i.e., roughly 20 km), the density retrieval can trend to the a priori assumption (see Evans et al. 2015) and potentially produce artifacts in retrieved CO_2_ density and derived temperature.

The same trends are noted for the outlier data points found in the temperature comparison between the two IUVS airglow datasets. As plotted in Fig. 6c, here the outlier data points are clustered primarily at cooler O I airglow-derived temperatures. As with the CO_2_ density, the RMA regression residuals are clustered at low altitudes, the southern hemisphere, high solar zenith angles, and at perihelion (and subsequently at high Lyman-$\alpha $ and global dust activity). From Table 2, the slope and intercept are significantly different than agreement conditions.

### IUVS O I 297 nm Airglow to IUVS Stellar Occultations

**Fig. 13:**
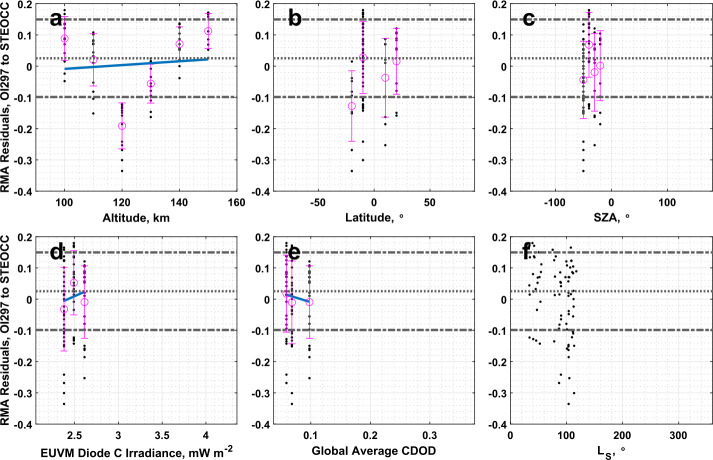
Residuals of the RMA regression between IUVS O I 297 nm airglow and IUVS stellar occultation CO_2_ density datasets plotted against (a) altitude, (b) latitude, (c) SZA, (d) EUVM diode C irradiance, (e) global average CDOD, and (f) $L_{\mathrm{S}}$. Red points indicate outliers. Magenta points with error bars indicate bin averages for the binning parameters and their standard deviations. The blue line indicates a linear fit to the binned data. Positive residuals indicate IVS O I 297 airglow data is measuring below the best-fit line

The CO_2_ density measured by the IUVS O I 297 nm airglow and stellar occultation datasets are very well correlated, with a correlation coefficient of 0.99 reported in Table 3. From Table 1, the slope is significantly different from one, and the intercept is significantly different from 0. Analysis of the residuals reveals an interesting structure in altitude as plotted in Fig. 13a, where the IUVS stellar occultations measure higher CO_2_ density than the O I airglow dataset at and around 120 km altitude. No structure in the residuals is noted in the other binning parameters.

**Fig. 14:**
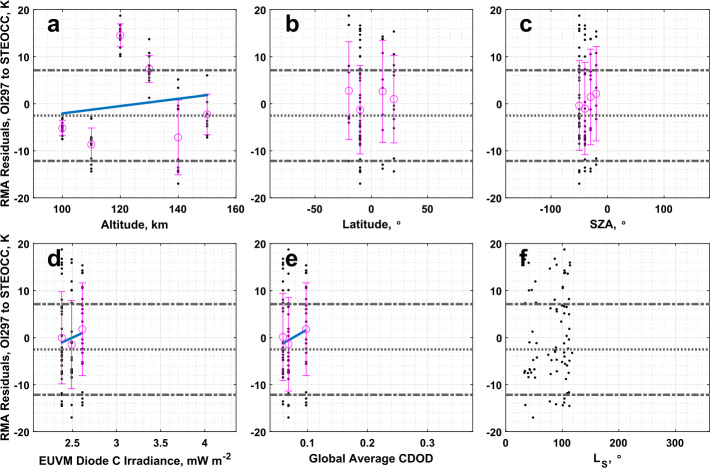
Residuals of the RMA regression between IUVS O I 297 nm airglow and IUVS stellar occultation temperature datasets plotted against (a) altitude, (b) latitude, (c) SZA, (d) EUVM diode C irradiance, (e) global average CDOD, and (f) $L_{\mathrm{S}}$. Red points indicate outliers. Magenta points with error bars indicate bin averages for the binning parameters and their standard deviations. The blue line indicates a linear fit to the binned data. Positive residuals indicate IUVS O I 297 airglow data is measuring below the best-fit line

A corresponding aberration in the residuals at 120 km altitude is noted in the comparison between the O I airglow and stellar occultation derived temperatures plotted in Fig. 14a, with similarly little structure in the residuals apparent in the other binning parameters. Table 2 records a slope and intercept significantly different from agreement conditions. It is worth noting that, unlike CO_2_^+^ UVD, which has a single emission peak, the O I 297 nm emission has two peaks, one around 130 km and a lower one around 90 km (Evans et al. 2023). There is a local minimum between the two peaks, which falls around 110-120 km. This local minimum represents a reduction in signal (i.e., information content) in the emission profile that may result in localized biases in the retrieved CO_2_ density and derived temperature.

### IUVS UVD Airglow to NGIMS

**Fig. 15:**
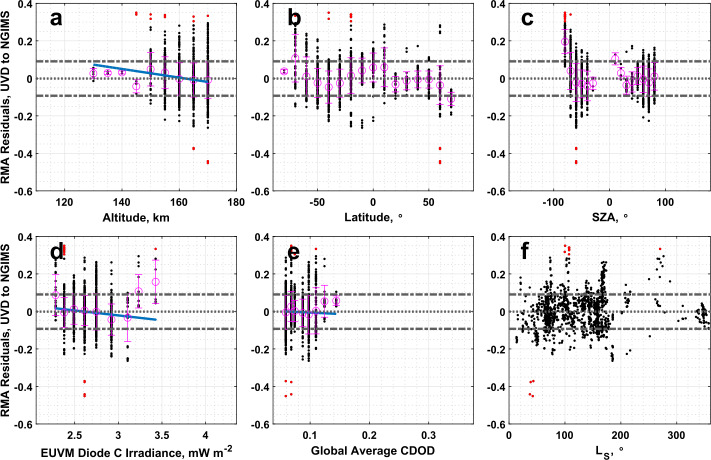
Residuals of the RMA regression between IUVS UVD airglow and NGIMS CO_2_ density datasets plotted against (a) altitude, (b) latitude, (c) SZA, (d) EUVM diode C irradiance, (e) global average CDOD, and (f) $L_{\mathrm{S}}$. Red points indicate outliers. Magenta points with error bars indicate bin averages for the binning parameters and their standard deviations. The blue line indicates a linear fit to the binned data. Positive residuals indicate IUVS UVD airglow data is measuring below the best-fit line

The comparison between CO_2_ densities of the IUVS UVD airglow and NGIMS datasets reveals them to be well correlated. The RMA regression slope and intercept recorded in Table 1 are outside $3\sigma $ from nominal agreement between the datasets. Some outlier data points are identified, occurring mainly at the dawn terminator in Fig. 15c. Weak, but statistically significant, correlations between the RMA regression residuals exist with altitude and Lyman-$\alpha $ irradiance.

**Fig. 16:**
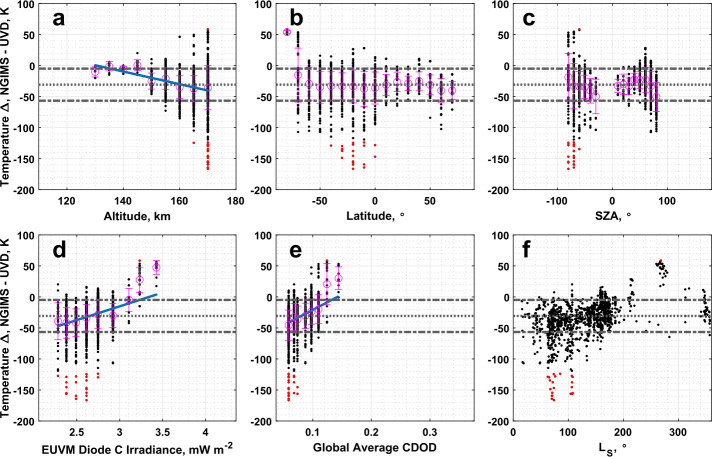
Binned temperature differences between IUVS UVD airglow and NGIMS temperature datasets plotted against (a) altitude, (b) latitude, (c) SZA, (d) EUVM diode C irradiance, (e) global average CDOD, and (f) $L_{\mathrm{S}}$. Red points indicate outliers. Magenta points with error bars indicate bin averages for the binning parameters and their standard deviations. The blue line indicates a linear fit to the binned data

The UVD airglow-derived temperatures are also poorly correlated with NGIMS temperatures, with a Pearson correlation coefficient of only 0.16 reported in Table 3. As with the solar occultation and O I airglow dataset, the UVD airglow data is warmer than NGIMS, though with a large spread in the data. Statistically significant correlations are reported for altitude, Lyman-$\alpha $ irradiance, and global average CDOD in Table 4. We note from Fig. 16a that the magnitude of the temperature difference appears to increase with altitude, while Fig. 16c shows the highest temperature differences are located at the dawn terminator. Examination of Fig. 16f shows that the largest negative temperature differences occur near aphelion, while the largest positive differences occur near perihelion. Figure 16b indicates that larger temperature disagreements occur in the southern hemisphere, though the highest, those identified as outliers, are clustered closer to the equator.

We recall from our discussion in Sect. 5.3 the issue of the UVD dataset trending towards its a priori assumptions at low altitudes when the UVD emission peak is elevated in altitude. This artifact does not appear in the comparison with the NGIMS datasets due to the inter-comparison not extending down to affected altitudes.

### IUVS UVD Airglow to IUVS Stellar Occultations

**Fig. 17:**
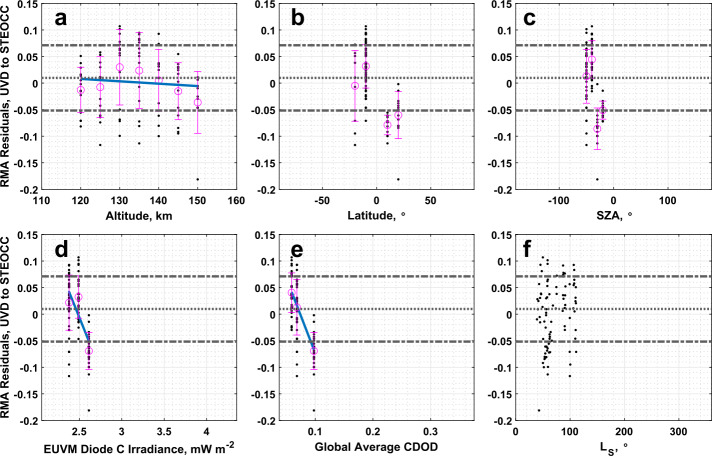
Residuals of the RMA regression between IUVS UVD airglow and IUVS stellar occultation CO_2_ density datasets plotted against (a) altitude, (b) latitude, (c) SZA, (d) EUVM diode C irradiance, (e) global average CDOD, and (f) $L_{\mathrm{S}}$. Red points indicate outliers. Magenta points with error bars indicate bin averages for the binning parameters and their standard deviations. The blue line indicates a linear fit to the binned data. Positive residuals indicate IUVS UVD airglow data is measuring below the best-fit line

The CO_2_ densities measured by the IUVS UVD and stellar occultation datasets show excellent correlation. The slope and intercept reported in Table 1 are significantly different from agreement conditions. Statistically significant correlations between the RMA regression residuals and Lyman-$\alpha $ irradiance and global average CDOD are reported in Table 4, however from Fig. 17d and Fig. 17e, we note that this comparison does not have extensive coverage in these two binning parameters, covering primarily low values corresponding to aphelion conditions. This limited coverage limits our confidence in these correlations.

**Fig. 18:**
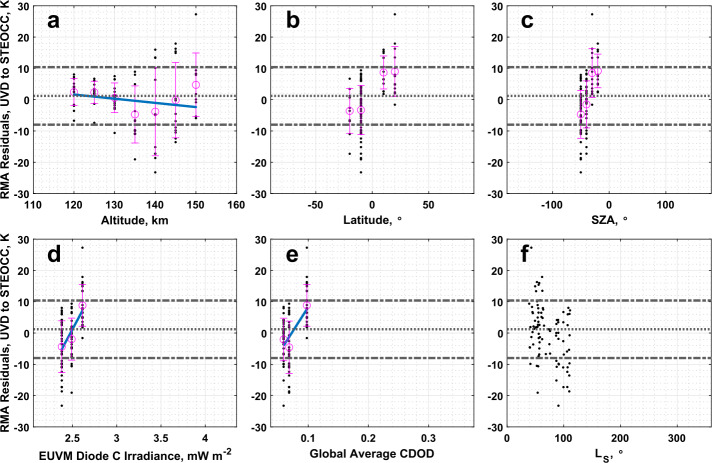
Residuals of the RMA regression between IUVS UVD airglow and IUVS stellar occultation temperature datasets plotted against (a) altitude, (b) latitude, (c) SZA, (d) EUVM diode C irradiance, (e) global average CDOD, and (f) $L_{\mathrm{S}}$. Red points indicate outliers. Magenta points with error bars indicate bin averages for the binning parameters and their standard deviations. The blue line indicates a linear fit to the binned data. Positive residuals indicate IUVS UVD airglow data is measuring below the best-fit line

Similar conclusions can be drawn for the comparison of the temperature data for the UVD airglow and stellar occultation datasets. However, we note that Table 2 records a slope and intercept for the RMA regression that are both within $3\sigma $ of agreement. We cannot demonstrate the UVD airglow and stellar occultation temperature datasets are in significant disagreement overall. Inspection of the residuals, plotted in Fig. 18 against various geophysical parameters, does not reveal any new structure besides that already noted from the density residuals.

As with the comparison of the UVD airglow dataset with NGIMS, the comparison of UVD airglow data with stellar occultations also does not appear to exhibit the a priori bias noted at low altitudes near perihelion that was prevalent in the comparison of UVD with O I 297 airglow results (Sect. 5.3). This is likely due to the inter-comparison of UVD and stellar occultation datasets not sampling perihelion conditions, and being restricted to aphelion (as illustrated in Fig. 17f and 18f).

### A Note on Dataset Adjustment

When adjusting the datasets using the adjustment parameters reported in Tables 1 and 2, it is important to realize that applying these adjustments breaks the link between density and temperature. The datasets utilized in this study derive their temperature estimates from density profiles through application of hydrostatic equilibrium. A density profile adjusted using Eq. (5), will not yield the same temperature profile as the original dataset or a temperature profile adjusted through Eqs. (6) or (7).

## Applications

In this section, we apply the corrections determined in Sect. 4 to the five datasets and derive additional products that illustrate the benefit of combining datasets for analysis. The post-adjustment products are compared with equivalent products constructed from the pre-adjustment datasets to compare the observed features and demonstrate the effects of applying the corrections to the datasets.

**Fig. 19:**
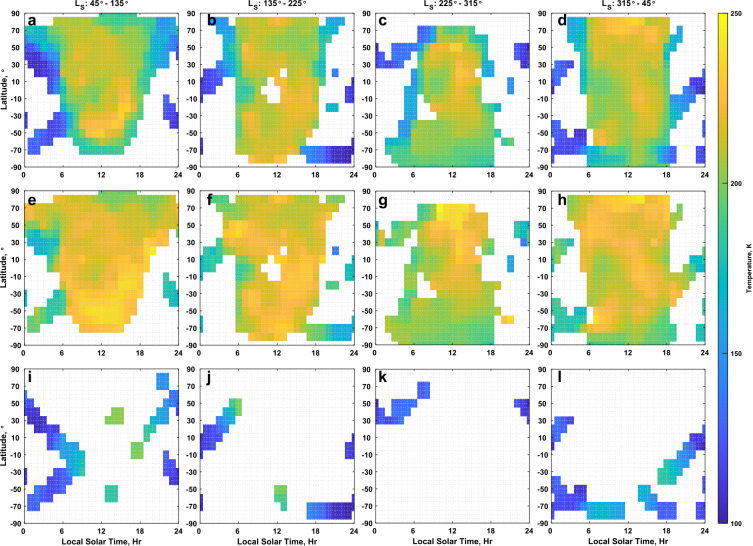
Maps of temperature at 150 km altitude in latitude and local solar time for southern hemisphere winter (a, e, and i) spring (b, f, and j), summer (c, g, and k), and fall (d, h, and l) pre- (a, through d) and post-adjustment (e through h) of the inter-comparison datasets. Datasets are adjusted to the O I 297 nm airglow dataset. The third row (i, j, k, and l) illustrates the coverage obtained at 150 km using only the NGIMS in-situ dataset with no adjustment applied

**Fig. 20:**
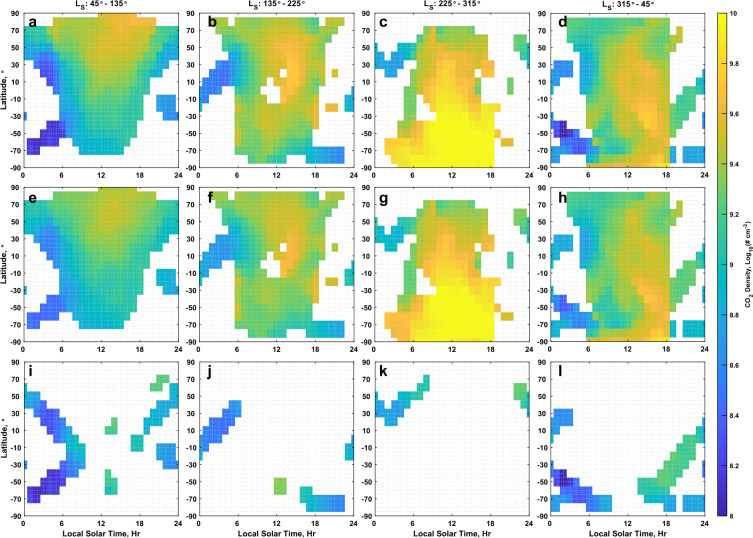
Maps of density at 150 km altitude in latitude and local solar time for southern hemisphere winter (a, e, and i) spring (b, f, and j), summer (c, g, and k), and fall (d, h, and l) pre- (a, b, c, and d) and post-adjustment (e, f, g, h) of the inter-comparison datasets. Datasets are adjusted to the O I 297 nm airglow dataset. The third row (i, j, k, and l) illustrates the coverage obtained at 150 km using only the NGIMS in-situ dataset with no adjustment applied

Figure 19 and Fig. 20 present maps of temperature and density, respectively, in latitude and local solar time for season at 150 km altitude. The maps were created by binning, with a weighting according to the measurement uncertainty, data from the five inter-comparison datasets by latitude (with bin centers placed every 10° and a bin width of 20°) and local solar time (with bin centers placed every hour and a bin width of 2 hours). Each column in Figs. 19 and 20 covers 90° in $L_{\mathrm{S}}$, with bin centers at 0°, 90°, 180°, and 270°. The figures presented average over the four Mars years utilized for the inter-comparison, from the start of MY 33 to the end of MY 36. The altitude of 150 km was chosen for this demonstration to maximize the contribution from each of the 5 datasets based on the altitude coverage shown in Fig. 1b. The datasets were adjusted to reference the O I 297 nm airglow dataset for the post-adjustment views presented in Fig. 19 and Fig. 20 to take advantage of the good correlation observed for both density and temperature between the IUVS datasets, while minimizing the impact of the poor correlation of NGIMS temperatures with the those of the other datasets. To adjust the EUVM solar occultation dataset to the O I airglow dataset, the solar occultation data was first adjusted to NGIMS, and then to O I, following the adjustment path laid out in Fig. 4. As part of the adjustment process, the uncertainties for the measurements were updated post-adjustment via Eqs. (8) through (10). The third row in Fig. 19 and Fig. 20 shows what the maps would look like only using the NGIMS in-situ dataset to illustrate the advantage of using multiple datasets.

From examination of the temperatures in Fig. 19, we observe several changes in the post-adjustment dataset. The first is that the temperature change from the nightside, primarily reported by NGIMS in these maps, to the dayside, primarily provided by the IUVS airglow datasets, has been reduced post-adjustment. In line with this, the atmosphere generally appears warmer, following the use of O I 297 nm airglow as the reference for the other datasets since this dataset tends to report warmer temperature than the others, as seen most easily by the higher temperature offset with NGIMS reported in Table 2. A clear signature of winter polar warming is seen in each temperature map of Fig. 19. However, in the pre-adjustment datasets maps, Fig. 19a and 19c, it appears limited to mid-latitudes, with higher latitudes being relatively cooler. This is a result of the EUVM solar occultation dataset reporting generally cooler temperatures than O I 297 nm airglow. Although these two datasets could not be compared directly due to a lack of overlap in solar zenith angle, by taking the solar occultation dataset through NGIMS to reach O I 297 nm airglow, we can correct for the apparent temperature discrepancy. In the post-adjustment maps, Fig. 19e and 19g, the temperatures remain high at higher latitudes, in agreement with previous observations of thermospheric polar warming (Bougher et al. 2006; Thiemann et al. 2024).

The differences in the pre- and post-adjustment density maps presented in Fig. 20 are more subtle and less extensive than for temperature, owing to the strong correlation that exists between the measured CO_2_ density of each dataset. Primarily, we note a reduced range of densities across local time that produces a smoother appearance to the density maps with less extreme gradients.

## Conclusion

Pairwise comparisons have been conducted between five MAVEN neutral atmosphere datasets for CO_2_ densities and neutral temperatures. This has revealed that while CO_2_ densities are well correlated between datasets, systematic differences do exist between them. In Table 1 we provide slope and intercepts for RMA regressions that can be used to adjust the publicly available MAVEN datasets to reference each other using Eqs. (5) and (8). In contrast, comparisons between NGIMS Ar temperatures are shown to be poorly correlated with EUVM solar occultation and IUVS airglow CO_2_ density derived temperatures. Median temperature differences have been determined to adjust datasets to and from an NGIMS reference. IUVS airglow and stellar occultation temperatures are well correlated, though like for CO_2_ density there are systematic differences, with the exception of UVD airglow and stellar occultation temperatures. Adjustment factors are reported in Table 2 for adjusting temperature datasets into nominal agreement. These factors can be used with Eqs. (6), (7), (9), and (10) to adjust the publicly available MAVEN datasets as performed in this paper.

## Data Availability

MAVEN IUVS O I 297 nm data are publicly available on CU Scholar (https://doi.org/10.25810/1BKN-BS85). The MAVEN/IUVS stellar occultation data are publicly available on CU Scholar (https://scholar.colorado.edu/concern/datasets/dz010s007). All other MAVEN data are publicly available at the MAVEN Science Data Center (https://lasp.colorado.edu/maven/sdc/public/) and the NASA Planetary Data System (https://pds-atmospheres.nmsu.edu/data_and_services/atmospheres_data/MAVEN/maven_main.html).
